# Estimation of the number of HCV-positive patients in Italy

**DOI:** 10.1371/journal.pone.0223668

**Published:** 2019-10-31

**Authors:** Ivan Gardini, Marco Bartoli, Massimiliano Conforti, Francesco Saverio Mennini, Andrea Marcellusi

**Affiliations:** 1 EpaC Onlus, Italian Liver Patient Association, Rome, Italy; 2 Centre for Economic and International Study (CEIS), Faculty of Economics, University of Rome "Tor Vergata", Rome, Italy; 3 Department of Accounting, Finance and Informatics, Kingston Business School, Kingston University London, London, United Kingdom; Centers for Disease Control and Prevention, UNITED STATES

## Abstract

**Background:**

HCV is one of the main causes of cirrhosis, hepatocellular carcinoma (HCC) and liver transplantation.

**Aim:**

The aim of this study was to estimate the number of living individuals diagnosed with hepatitis C in Italy. This study also aimed to stratify these subjects as diagnosed and cured, diagnosed awaiting a cure, and undiagnosed (individuals who were not diagnosed, living or lived with hepatitis C).

**Methods:**

To quantify the number of ill patients in Italy, an inquiry was conducted based on questionnaires submitted to three nationally representative regions, namely, Campania, Lazio and Piemonte, as representatives of the three main areas of Italy (North, Centre and South regions). The data were collected through a questionnaire to acquire demographic and clinical information on patients in the participating hospitals. The questionnaires contained 6 questions on sex, age, region of residence, disease condition, type of exemption and category. The questionnaires were administered individually to consecutive patients through face-to-face interviews conducted by specialised personnel in each centre. Data were collected between September 2017 and January 2018.

**Results:**

In total, 2,860 questionnaires were analysed. They were completed by the patients (55% male), who had an average age of 61 years (64 years for women and 59 years for men). In total, 54% of the sample declared that they were still infected with HCV (1,548 patients out of 2,860 respondents), while the remaining subjects declared that they had been cured. The inquiry showed that 46.6% of the sample had at least a 016 exemption (chronic hepatitis), while more than 51% (1,469 interviewed patients out of 2,860 respondents) had a different type of exemption. Only 2% of the respondents declared that they had no exemption. Assuming that the analysed sample is representative of the actual HCV-positive population in Italy and considering the number of 016 exempt patients in the regional data, the model estimates that there are 443,491 cured and HCV-positive living patients and 240,043 ill patients who have yet to be treated.

**Conclusions:**

Although this study has limitations, it represents a considerable improvement over the previously available studies. This study can help decision-makers implement more effective strategic planning to eliminate hepatitis C.

## Introduction

Hepatitis C is a liver infection caused by the hepatitis C virus (HCV). HCV is the main cause of cirrhosis, hepatocellular carcinoma (HCC) and liver transplantation [1–3]. In most cases, the infection is spread through parenteral transmission or by blood-to-blood contact. It is transmitted less frequently by sexual transmission and very rarely by vertical transmission (from mother to child) [4, 5]. The aim of the World Health Organization is to eliminate the HCV virus by 2030 [6].Any intervention programme aimed at eliminating the disease must be based on key information, specifically the estimated number of patients suffering from this disease who have or have not been diagnosed [7].

However, there is no registry of patients diagnosed with hepatitis in Italy. The estimated epidemiological data are based on information from the 1990s [2, 8–10] and cannot be considered reliable as the epidemiological scenario has completely changed due to new treatments that have cured many patients and to deaths associated with age, complications and comorbidities.

In 2015, a study conducted with EpaC Onlus patients in collaboration with the Economic Evaluation and Health Technology Assessment (EEHTA) group at the University of Rome “Tor Vergata” [11] attempted to estimate the number of diagnosed patients who had yet to be treated based on evidence from the literature and the available regional exemption registers in Italy. In this study, the number of diagnosed patients who still need to be treated was estimated based on data on exempt patients classified with the sub-code 016 (Chronic Hep C) obtained from the regional and local health authorities. However, the global data regarding exempt patients was not sufficient to quantify the total number of ill patients who used a different exemption or did not benefit from any exemption at all. Furthermore, due to delays in the updating of the exemption registers, some exempt patients could already be cured or dead but would still be listed on the registry. The number of cured patients was estimated based on data collected with a questionnaire submitted to the EpaC patients and the number of patients who benefitted from other exemption codes or who did not benefit from any exemption. Overall, the study estimated that more than 430,000 subjects had contracted the disease, of whom approximately 200,000 were HCV positive as of 2015 [11].

The main limitation of the 2015 study concerned the source of the information used to process the exemption data. This was because the EpaC patients were more aware of their health status and rights, generating a selected and thus only partially representative population.

Following this research, the aim of this study was to estimate the number of living individuals diagnosed with hepatitis C in Italy. This study also aimed to stratify these subjects as diagnosed and cured, diagnosed awaiting a cure, and undiagnosed (individuals who were diagnosed, living or lived with hepatitis C).

## Materials and methods

To quantify the number of HCV-positive patients in Italy, an inquiry was conducted based on a specific questionnaires administered in three Italian regions, namely, Campania (Evangelico Villa Betania Hospital, Naples; Hospital of Gragnano, Gragnano (NA)), Lazio (University Hospital Umberto I, Rome; University Hospital Tor Vergata, Rome; Villa Maraini Foundation for Addicted People, Rome) and Piemonte (University Hospital Città della Salute e delle Scienze, Turin; Hospital Amedeo di Savoia, Turin; Hospital Madonna del Popolo di Omegna (VB); University hospital San Luigi Gonzaga, Orbassano (TO); Hospital SS Antonio e Margherita, Tortona (AL); Hospital Ordine Mauriziano—Umberto I, Turin; University hospital Maggiore della Carità, Novara). These three regions were selected as representative of the three main areas of Italy (North, Centre and South regions).

The analysis was conducted through a questionnaire that collected demographic and clinical information about the hospitalised patients. The questionnaire was composed of 6 questions about sex, age, region of residence, disease condition (infected or cured before and after 2015), type of exemption and category (HCV mono-infected, HIV/HCV co-infected, active people who use drugs, drug addict undergoing replacement therapy). The survey was conducted by the health personnel of the public hospitals authorised to prescribe new direct-acting antivirals (DAAs) and centres specialising in managing these patients. The questionnaires were administered to consecutive patients during face-to-face interviews conducted by specialised personnel in each centre. Inmates and non-European citizens without a residence permit were not included in this study. Data were collected between September 2017 and January 2018.

We considered the number of exempt patients (coded 016) estimated in the study by EpaC/EEHTA in 2015[11] and assumed that during the following 3 years, 15,000 subjects died [12]. Patients with a 016 exemption (Chronic Hep C) have the ability to save money on the visits and exams (related to the specific disease) that become free for those who have the exemption. All patients with a diagnosis of Hep C can ask for this exemption with a specific certificate provided by a specialist. These registry data were the only data at the national level. Starting from these data, we proportionated all the data derived from the questionnaire to obtain an inference estimation. Thus, our sample was assumed to be representative of the population (the main important and populated region for each Italian geographical area). The sample was classified as ill or cured patients, ill exempt or not exempt patients, and not ill exempt or not exempt patients. The estimation was based on the reproportioning of exempt patients in Italy, applying the weights calculated based on the results of the inquiry. In other words, the number of subjects in Italy for each group analysed (diagnosed and cured, diagnosed awaiting a cure, and undiagnosed with or without exemptions) were estimated considering the stratification derived from the sample applied proportionally to the total number of exemptions in Italy derived from the literature [11]. Each group correspond to a specific question on the questionnaire.

A deterministic sensitivity analysis (DSA) was conducted to account for the potential selection bias due to the smaller number of cured patients who presumably visit specialised centres less often than patients currently undergoing treatment (or those who have been recently cured). The DSA uses a one-way deterministic approach (one parameter changed for each simulation) with the results obtained by changing one parameter of the model at a time according to the variability found in the literature or assumed by the authors. In this case, the DSA was conducted assuming the following variations:

- exempt and not exempt patients: ± 20% versus the baseline case;
- exempt patients: ± 20% versus the baseline case; and
- diagnosed patients: ± 20% versus the baseline case.

## Results

### Sample characteristics

In this study, 2,860 questionnaires were analysed. The questionnaires were completed by the patients (55% male), who had an average age of 61 years (64 years for women and 59 years for men). In Table 1, we report the sample characteristics.

**Table 1:** Descriptive statistics of the sample stratified by region (Total sample N = 2,860).

Distribution by age, sex and region	CAMPANIA	LAZIO	PIEMONTE	Total
Total sample N	824	600	1,436	2,860
% Males	46.4%	62.3%	56.4%	54.8%
Average age of the sample	65.7	59.3	58.9	61.3
Regional distribution by type of disease	CAMPANIA	LAZIO	PIEMONTE	Total
Viremic patients N (%)	509 (61.8%)	260 (43.3%)	779 (54.2%)	1,548 (54.1%)
Pre-2015 SVR N (%)	18 (2.2%)	10 (1.7%)	97 (6.8%)	125 (4.4%)
Post-2015 SVR N (%)	297 (36.0%)	330 (55.0%)	560 (39.0%)	1,187 (41.5%)
Type of exemption by disease	CAMPANIA	LAZIO	PIEMONTE	Total
016 Exemption N (%)	221 (26.8%)	213 (35.5%)	537 (37.4%)	971 (34.0%)
016 Exemption+others N (%)	37 (4.5%)	21 (3.5%)	303 (21.1%)	361 (1.6%)
Other exemptions N (%)	539 (65.4%)	356 (59.3%)	574 (40%)	1,469 (51.4%)
No exemption N (%)	27 (3.3%)	10 (1.7%)	22 (1.5%)	59 (2.1%)
Regional distribution of co-infected patients	CAMPANIA	LAZIO	PIEMONTE	Total
HCV mono-infected N (%)	822 (99.8%)	576 (96.0%)	1,251(87.1%)	2,649 (92.7%)
HCV-HIV co-infected N (%)	0	24 (4.0%)	183 (12.7%)	207 (7.2%)
HCV-HBV co-infected N (%)	2 (0.2%)	0	2 (0.1%)	4 (0.1%)
Regional distribution of people who use drugs	CAMPANIA	LAZIO	PIEMONTE	Total
HCV mono-infected N (%)	816 (99.1%)	349 (58.2%)	1,151 (80.2%)	2,316 (81.0%)
HCV ExTD mono-infected N (%)	6 (0.7%)	0	41 (2.9%)	47 (1.6%)
HCV+HBV N (%)	2 (0.2%)	0	2 (0.1%)	4 (0.1%)
HCV+HIV N (%)	0	2 (0.3%)	161 (11.2%)	163 (5.7%)
HCV+HIV+ exTD N (%)	0	0	14 (1.0%)	14 (0.5%)
HCV+HIV+TD N (%)	0	22 (3.7%)	8 (0.6%)	30 (1.0%)
HCV+TD N (%)	0	227 (37.8%)	59 (4.1%)	286 (10.0%)

SVR: sustained virological response

Overall, 54% of the sample declared they were still HCV positive (1,548 patients out of 2,860 respondents), while the remaining part stated that they had been cured. This result was confirmed by data at the regional level, with 38% in Campania (315 out of 824 respondents) and 56.8% in the Lazio region (340 out of 600 respondents). Presumably, most part of the sample were cured in the post-2015 period (125 vs. 1,187) (Table 2).

**Table 2:** Sample distribution based on 2,860 interviews–Number of subjects for each group, percentage distribution by row and column.

A–Absolute values			
	Total Sample (N)	016 Exempt (N)	016 Not exempt (N)
Overall	2,860	1,332	1,528
Ill	1,548	718	830
Not ill	1,312	614	698
B–Percentage distribution of exempt and non-exempt patients			
	Total Sample	016 Exempt	016 Not exempt
Overall	100%	47%	53%
Ill	100%	46%	54%
Not ill	100%	47%	53%
C–Percentage distribution of ill and not ill			
	Total Sample	016 Exempt	016 Not exempt
Overall	100%	100%	100%
Ill	54%	54%	54%
Not ill	46%	46%	46%

Furthermore, the inquiry showed that 46.6% of the sample population had at least a 016 exemption (chronic hepatitis), while more than 51% (1,469 interviewed patients out of 2,860 respondents) had a different exemption. Only 2% of the respondents declared that they had no exemption (Table 1).

More than 92% of the subjects in the sample were HCV mono-infected, while slightly more than 7% were co-infected with HCV and HIV, and only 0.1% were co-infected with HBV and HCV (Table 1). With regard to the part of the sample addicted to drugs, 377 interviewed patients (13.8% of the sample) claimed they were currently experiencing or had previously experienced drug addiction (61 ex-drug user), and 211 respondents (7.3% of the sample) had co-infections with HCV and HBV or HIV (Table 1).

### HCV patients with known diagnoses

Table 2 shows the main results of the inquiry with reference to the patients’ conditions (ill and not ill) and the presence of a 016 exemption. Out of 2,860 interviewed patients, 47% of the subjects had a 016 exemption (Table 2B). Of these, only 54% were still ill (Table 2C). Similarly, 53% of the interviewed subjects did not have a 016 exemption (Table 2B), even if they were still ill (Table 2C).

**Fig 1:**
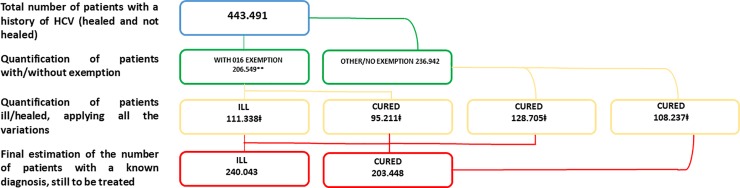
Results of the estimation of the HCV-positive population with known diagnoses–EpaC inquiry into 2018 exemptions.

Fig 1 shows the results in the HCV population with a known diagnosis estimated by the inference model. The model estimated 443,491 total cured and viremic living patients and 240,043 ill patients who had yet to be treated. This was based on the assumption that our sample was representative of the real HCV-positive population in Italy and that the number of patients with a 016 exemption in the regional data could be extrapolated to the general population.

*Population obtained from the regional database of exemptions: HCV-positive population at the beginning of 2015 with 016 exemptions corrected based on the assumption of 15,000 estimated deaths (50% of the total) between 2015 and 2018 (ISTAT). *‡* Population obtained from the EpaC 2017 questionnaire with 2,860 HCV-positive patients in specialised medical centres. This population is more representative of the HCV-positive population currently residing in Italy (inmates and non-European citizens without a residence permit were not included in this study). In this investigation, co-infected patients and *people who use drugs* were included.

### Sensitivity analysis

**Fig 2:**
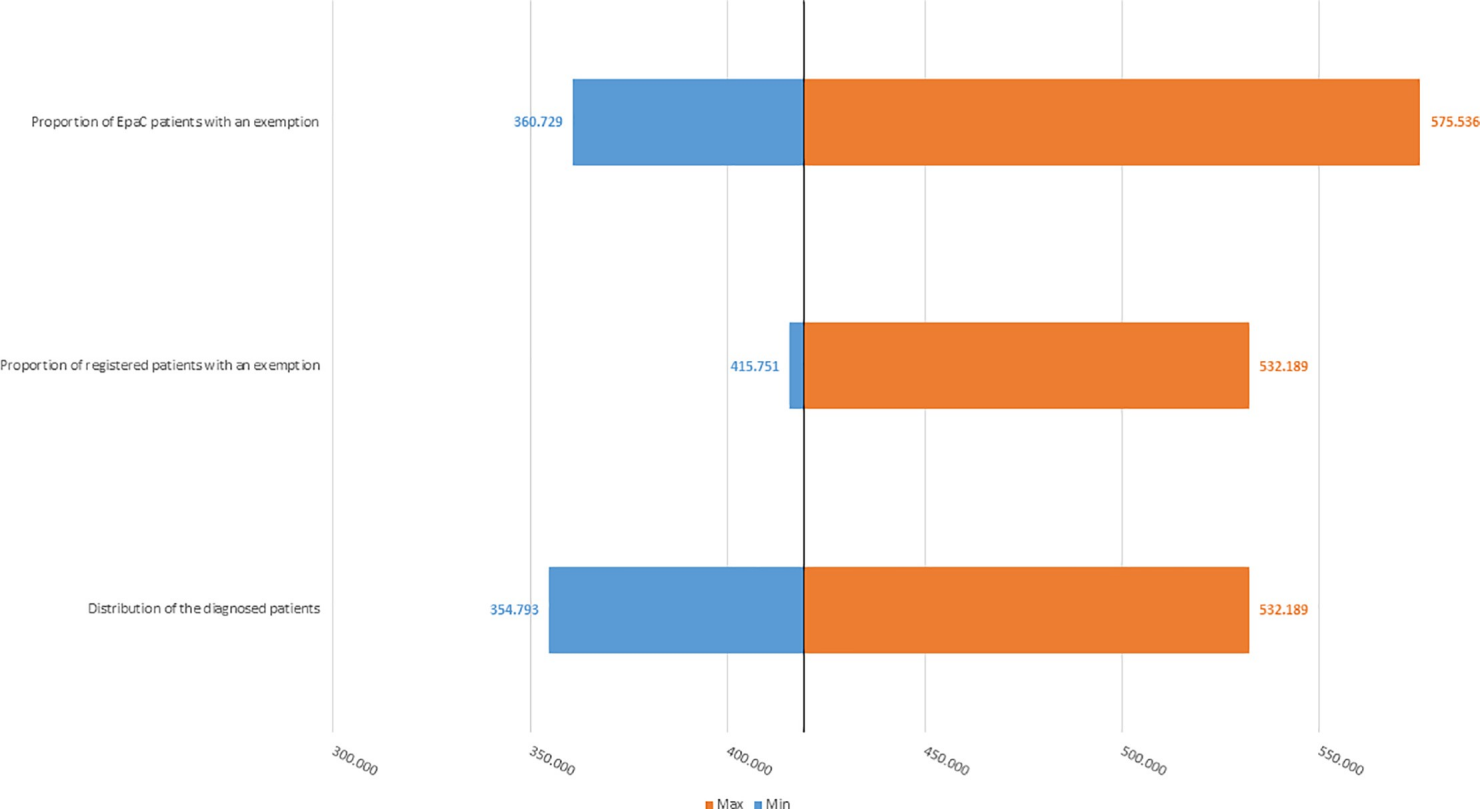
One-way deterministic sensitivity analysis–estimated number of HCV-positive patients estimated for the base-case analysis (black-line) and each simulated scenario (± 20%).

**Fig 3:**
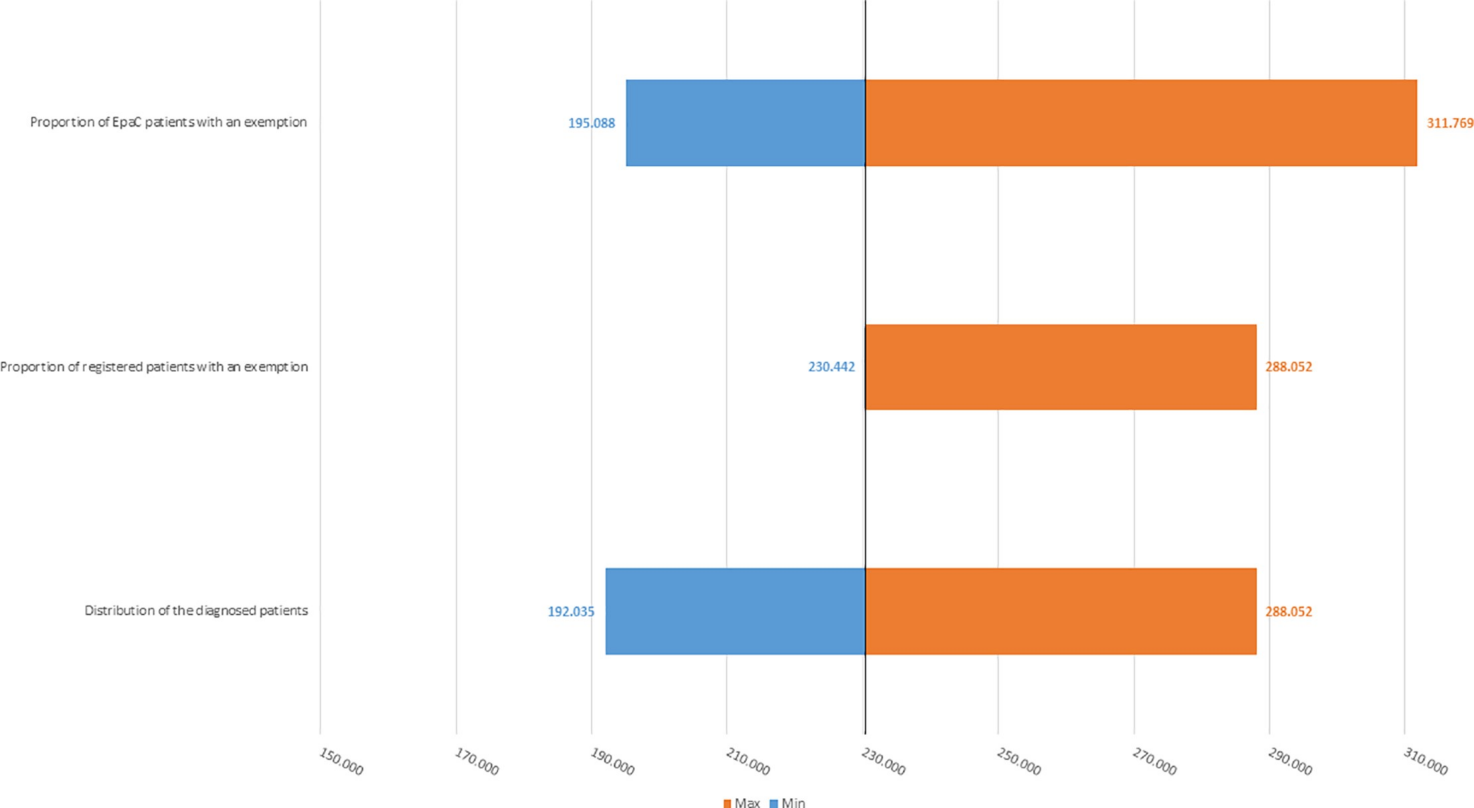
One-way deterministic sensitivity analysis–estimated number of HCV-infected patients who had yet to be treated as of 1 January 2018 estimated for the base-case analysis (black-line) and each simulated scenario (± 20%).

Fig 2 shows that based on the scenarios previously reported, the number of HCV-positive patients may range from a minimum of 354,793 to a maximum of 575,536 patients (baseline case: 443,491 patients). Fig 3 shows that the number of ill patients who have yet to be treated may range from a minimum of 192,035 to a maximum of 311,793 patients (baseline case: 240,043 patients).

## Discussion

This study estimated the total number of patients diagnosed with HCV in Italy and quantified the number of subjects who still need to be treated. The inquiry attempted to overcome the critical issues in the prevalence studies conducted in the 1990s and 2000s [11] and to provide updated estimates than can be used by healthcare decision-makers to adequately plan for the elimination of HCV.

Based on the previous study performed in 2015 [11], the model estimated a reduction in the percentage of patients with 016 exemptions in the total HCV population (72% in 2015 vs. 47% in 2018). Moreover, compared with the 2018 study, the 2015 study estimated a lower number of cured subjects (20% in 2015 vs. 46% in 2018). However, this estimation is consistent with the high number of patients treated during the last 3 years [13].

This analysis updates the estimate of the total prevalence of HCV infections, including the subgroups of viremic patients who have yet to be treated, and it provides some indications regarding the characteristics of these patients. To implement appropriate prevention strategies, it is necessary to identify viremic individuals with high-risk behaviours. However, according to the literature and reports by public institutions, the estimates of the numbers of HIV/HCV co-infected subjects, people who use drugs and inmates within the HCV-positive population are highly variable as there is a lack of information (e.g., drug users) and the dynamics are complex (e.g., detention mobility).

With regard to the population of HIV/HCV co-infected patients, based on the information obtained from the ICONA register (“ICONA” Italian cohort of HIV-infected patients) [14–16] and assuming that a conservative 10% of HCV-positive subjects are co-infected with HIV (presentation by Professor Massimo Galli, Vice President of SIMIT, 5 July 2017), it is possible to obtain an estimate of approximately 9,000 HIV/HCV co-infected patients yet to be treated as of 2018. Moreover, our investigation estimated that approximately 13% of HIV/HCV co-infected patients have 016 exemptions.

In relation to the population who use drugs patients, according to the data in the last Drug Consumption Report (2015) [17], 461,500 individuals have problems with drug addiction, only 143,271 of whom are followed in public hospitals (SerD), as described in the 2016 parliamentary report [18]. The data in the parliamentary report indicate that 28,197 of these individuals received HCV tests, corresponding to 20.5% of the total subjects followed by hospitals and 27.0% of those who used injected drugs. Other published sources [19] estimate that 68% of drug users, particularly heroin users, are infected with HCV.

The estimates in the Parliamentary Annual Reports of 2008 [20] and 2011 [21] indicated similar HCV positivity estimates among drug users of 65% and 61%, respectively. Further information can be found in the “DTPI Project–Diagnosis and early therapy of drug-related infections” [22], which collected data in 9 drug addiction centres in 5 different cities and included approximately 400 drug users. The prevalence of HCV infection was 20.4%. To summarise, applying the abovementioned 2015 Parliamentary Report estimate to the total number of people who use drugs (461,500 subjects), we obtained an HCV infection rate of 9%, corresponding to approximately 41,500 HCV-infected subjects. Conversely, if we refer to the total number of injection/inhalation drug users, which amounts to 92,300 subjects with an estimated HCV infection rate of 28–30%[17], we obtain approximately 27,000 subjects.

Prison inmates represent another population at high risk of infection. The study by ARS Toscana [23], which is the most important work that has been conducted on the subject, involved 57 penitentiaries and 15,000 people and estimated an HCV prevalence rate of 7.4% among the inmates. The study was based on the evaluation of the medical records of inmate patients and did not take into account additional diagnostic interventions, which means that there was a risk of underestimating the real prevalence of HCV infection among the inmates. Studies conducted by testing inmates reported prevalence rates of 38.8% [24] and 22.8% [25]. According to the report by the Ministry of Justice dated 31 May 2018 [26], the actual number of inmates is approximately 58,500. Applying the prevalence rates to this number of subjects, the number of HCV-infected inmates can be estimated to be between 4,000 and 16,000.

Finally, to eradicate the virus, an important debate has been held in recent years regarding the number of diagnosed incident HCV patients in Italy. With reference to new HCV infections, according to the Integrated Epidemiologic System of Acute Hepatitis, new cases of HCV were observed in 0.18 x 100,000 inhabitants in 2016 (SEIEVA) [27, 28]. In 2013, the same estimates indicated a rate of 0.27/100,000 inhabitants, which means that over the last 4 or 5 years, there was a decrease in the prevalence of new HCV infections, reaching 0.2–0.3/100,000 inhabitants. Given that the literature indicates that acute hepatitis caused by HCV occurs in an estimated 7% and 15% of all infected subjects [29], it is possible to estimate that approximately 1,100 new infections occur every year in Italy. Of these, we can assume that only 25–30% of people spontaneously eliminate the virus and are never diagnosed [30].

A different issue is the estimate of the population with non-acute and undiagnosed HCV infections. The number of these hidden cases can be estimated by determining the size of the sub-population at high risk of infection. To estimate the prevalence in the general population under 65 years of age without additional risk factors, we used the information contained in the ISTISAN reports of the Institute of Health related to infections identified among blood donors [31].

In total, 0.013–0.05% of HCV-infected subjects were identified among 1.9 million tested people. When applied to the Italian population under the age of 65 years, this rate yields an estimate of no more than 15,000 individuals. With reference to the Italian population over the age of 65 years, the most recent prevalence estimates in the literature (Andriulli et al. [32] and di Morisco et al. [33]) indicate that 20% of the total HCV-positive population has undiagnosed infections. Applying this value proportionally to the number of diagnosed subjects estimated in our study, we can estimate that the number of undiagnosed subjects over the age of 65 years in Italy ranges between 35,000 and 57,000 people.

In summary, including the hidden patients previously discussed, we can assume that there are between 66,000 and 130,000 undiagnosed patients. Of these, more than 50% are concentrated in the subgroup of the population who are at risk for HCV infection (drug users, inmates and people over the age of 65 years).

Clearly, our inquiry has some limitations that should be taken into account when making policy decisions. First, the subjects who replied to the inquiry regarding exemptions were patients who regularly attended authorised centres during their pre- and post-treatment process. Consequently, the number of patients not followed or partially followed in authorised centres due to their recovery may have been underestimated. A second limitation is the mixture of information used to estimate the number of patients diagnosed at the national level. The estimation was based on the number of exempt patients at the national level, which may represent a confounding factor as this value was not updated in 2018 (the data are from 2015) and the database is not immediately updated at the regional level.

## Conclusions

Starting from the limitations and critical issues of the previous study in 2015 [11], this work attempted to estimate the number of living patients with a history of HCV in Italy, including diagnosed and undiagnosed cured and viremic patients, to update the estimation of the number of patients who have yet to be treated.

This analysis yielded an estimate of 443,491 cured patients, viremic patients or patients with a history of HCV. This figure is higher than that obtained by the previous analysis, which estimated 308,624 subjects. With reference to the number of ill patients yet to be treated as of 1 January 2018, this analysis estimated 240,043 subjects, which is higher than the figure estimate in the 2015 study, taking into account the number of patients who were cured or who died in the last 3 years. However, the figure is lower than the historical prevalence rate in Italy, which was more than 1.5%.

This study suggests an important strategy that could be implemented to eradicate HCV: patients who need to be treated are more likely to be found outside of authorised public hospitals than in them. Specifically, patients who do not attend authorised centres, such as drug users, inmates or those belonging to the microbasins (transfusion dialysis centres [haemophilia thalassaemia], dialysis centres, foster homes, drug rehabilitation centres), should be identified by unauthorised centres or general practitioners.

Furthermore, this study proposes the first estimation of the number of people with undiagnosed (hidden) HCV infections through a separate analysis of high-risk groups and different age groups in the general population. This estimation provides important information on the possible localisation of patients who have yet to be diagnosed and what the main objectives of intervention programmes should be, such as screening, information and prevention. Immigrants illegally living in Italy were excluded from the analysis given their high degree of mobility and the lack of epidemiological data. However, research was conducted to collect currently available information.

To implement a treatment plan to eradicate the virus, it is necessary to quantify infected patients and to have a clear idea of their distribution and of the methodology that should be used to identify and treat them. An adjusted and shared micro- and macro-PDTA to treat and follow the patients should be activated, keeping in mind that each subgroup of patients is associated with different basins and stakeholders. Any national or local elimination plan should take into account all the existing realities and should communicate and cooperate with other stakeholders when planning the strategy to be adopted [2, 34].

In conclusion, the statistical analysis of the data allowed us to update the estimation of the epidemiological scenario of patients with known HCV diagnoses. Therefore, this work represents a considerable improvement over the 2015 study, and it is a useful tool for decision-makers who seek to implement a more effective strategic plan to treat and eradicate the virus.
